# Profile of patients treated with intravitreal antiangiogenics in a Brazilian public service with high level of complexity

**DOI:** 10.5935/0004-2749.2022-0119

**Published:** 2023-03-20

**Authors:** Isadora Andrade Rabelo, Marina Crespo Soares, Andrea Mara Simões Torigoe

**Affiliations:** 1 Serviço de Oftalmologia, Hospital das Clínicas, Universidade Estadual de Campinas, Campinas, SP, Brazil.

**Keywords:** Retina, Intravitreal injections, Angiogenesis inhibitors, Macular edema, Retinal neovascularization, Retina, Injeções intravítreas, Inibidores da angiogênese, Edema macular, Neovascularização retiniana

## Abstract

**Purpose:**

Intravitreal antiangiogenic therapy is currently the most invasive ophthalmic procedure performed worldwide. This study aimed to describe the clinical and epidemiological profile of patients undergoing intravitreal antiangiogenic therapy in a tertiary referral hospital in Brazil.

**Methods:**

This cross-sectional, retrospective, and observational study analyzed medical records of patients who received intravitreal injections of antiangiogenic agents for the treatment of retinal diseases at the ophthalmology outpatient clinic in the Hospital das Clínicas at Unicamp between January and December 2020.

**Results:**

The study included 429 patients and 514 eyes. The study population was predominantly male (51.28%), white (80.89%), between 50 and 80 years old (mean age, 60.92 years), had complete or incomplete first-grade education (56.88%), and did not belong to the Regional Health Department of which Campinas is a part (78.55%). Bevacizumab was the most commonly used intravitreal injectable medicine (79.38%), pro re nata was the most commonly used treatment regimen (90.27%), and macular edema was the most prevalent pathology indicative of treatment (60.12%), with diabetes etiology accounting for 48.25%. The average number of injections per patient was 3.83, with the macular neovascularization group and the pro re nata group having the highest and lowest with five and three injections, respectively. Treatment adherence was associated with the patient’s pathology, and the macular edema (52.24%) and macular neovascularization (49.48%) groups had the lowest adherence rates.

**Conclusions:**

This study evaluated the epidemiological and clinical profile of patients undergoing antiangiogenic therapy in a high-complexity public hospital, which is fundamental for a better understanding of the demand for ophthalmic reference service in Brazil, and the analysis of functional results and user adherence profile promotes optimization of indications and leverages the benefits of intravitreal therapy.

## Abstract (pt)

**Objetivo:**

A terapia antiangiogênica intravítrea revolucionou o tratamento de inúmeras patologias de relevância global, sendo atualmente o procedimento oftalmológico invasivo mais realizado no mundo. Objetiva-se no presente estudo descrever o perfil clínico e epidemiológico dos pacientes submetidos a terapia intravítrea com antiangiogênicos em hospital terciário de referência no Brasil.

**Métodos:**

Trata-se de um estudo transversal, retrospectivo e observacional que foi realizado através da análise de prontuários de pacientes submetidos a injeção intravítrea de antiangiogênicos para tratamento de doenças retinianas no ambulatório de oftalmologia do Hospital das Clínicas da Unicamp no período de janeiro a dezembro de 2020.

**Resultados:**

O estudo analisou 429 pacientes e 514 olhos. A maioria pertencia ao sexo masculino (51,28%), raça branca (80,89%), possuía entre 50-80 anos com idade média de 60,92 anos e escolaridade de 1º grau completo ou incompleto (56,88%) e não pertenciam (78,55%) a área de abrangência do Departamento Regional de Saúde do qual Campinas faz parte. O fármaco mais utilizado nas injeções intravítreas foi o bevacizumabe (79,38%), o principal regime de tratamento foi o pro re nata (90,27%) e a principal grupo de patologia indicativa de tratamento foi o edema macular (60,12%), sendo 48,25% desses de etiologia diabética. A média de injeções foi de 3,83/paciente, sendo o grupo de neovascularização macular o de maior mediana com 5 injeções/paciente e o esquema pro re nata o regime de tratamento com menor mediana, 3 injeções/paciente. A adesão ao tratamento associou-se a patologia do paciente, sendo as menores taxas de adesão as dos grupos com edema macular (52,24%) e neovascularização macular (49,48%).

**Conclusões:**

O presente estudo avaliou o perfil epidemiológico e clínico dos pacientes submetidos a terapia antiangiogênica em hospital público de alta complexidade, o que é fundamental para melhor conhecimento da demanda de serviço oftalmológico de referência no Brasil e possibilita, a partir da análise dos resultados funcionais e perfil de adesão dos usuários, otimizar as indicações e alavancar os benefícios de terapia intravítrea.

## INTRODUCTION

Intravitreal injection is the most performed invasive ophthalmic procedure worldwide. Its use has led to a breakthrough in the treatment of diseases of worldwide relevance, and the estimated number of injections in the United States had increased from less than 3,000 per year in 1999 to approximately 6.5 million in 2016^(1,2)^.

Antiangiogenic therapy targets an extracellular protein called vascular endothelial growth factor (VEGF). This protein is a significant mediator of vascular growth and acts with an increase in vascular permeability^(3,4,5)^.

Anti-VEGF treatment is indicated for several retinal pathologies, mainly diabetic macular edema (DME) that is caused by altered retinal vascular permeability, leading to an increase in retinal thickness, formation of intraretinal cysts located mainly in the plexiform layers (external and internal nuclear layers), and neovascular age-related macular degeneration (AMD), which is associated with neovascular macular membrane formation with secondary exudation.

The main signs of macular neovascularization (MNV) activity are hemorrhage on funduscopy, leak on fluorescein angiography or presence of subretinal or intraretinal fluid, lipid exudates, and subretinal hyperreflective material (SHRM) on optical coherence tomography. Clinically, when active, these diseases usually manifest with reduced visual acuity (VA) and scotomas and metamorphopsia in the presence of MNV^(6,7,8,9,10,11,12,13,14,15)^.

Several anti-VEGF drugs with different mechanisms are available. Bevacizumab (Avastin®) is a monoclonal antibody that has been approved by the Food and Drug Administration (FDA) for the treatment of colorectal cancer, but is widely used off-label in ophthalmology because of its cost-effectiveness. Ranibizumab (Lucentis®), an antibody fragment that inhibits all isoforms of VEGF-A, and Aflibercept (Eylea®), a fusion protein designed to bind to VEGF-A, VEGF-B, and platelet growth factor, were both FDA approved for the treatment of AMD in 2007 and 2011, respectively^(16,17,18)^.

Given the significant results obtained with intravitreal anti-VEGF treatment in the literature, this study aimed to describe the clinical and epidemiological profile of patients undergoing intravitreal therapy with antiangiogenic agents in a tertiary referral hospital in Brazil to gain more knowledge about the treated population and the use of this therapy in the Brazilian Unified Health System (SUS).

## METHODS

This cross-sectional, retrospective, and observational study analyzed medical records of patients who received intravitreal injections of antiangiogenic agents in the treatment of retinal diseases at the ophthalmology outpatient clinic in the Hospital das Clínicas at Unicamp, Campinas City, SP, Brazil, between January and December 2020.

This study was approved by the Research Ethics Committee of the State University of Campinas (Certificate of Submission for Ethical Appreciation -CAAE no. 46509821.1.0000.5404), following the precepts of the Helsinki Declaration and the Nuremberg Code.

All patients aged >18 years who agreed to participate in the study by signing a free and informed consent form and were contacted by telephone were included. Those who died during the study period or whose medical records were lost were excluded.

The following patient identification variables were analyzed: age, sex, education, race, origin, point of entry into the service, and clinical history, including information about the pathology indicating the treatment, anti-VEGF drug used, number of injections, treatment regimen, change in treatment (switch), missed appointments for intravitreal injections, outcomes, and change in VA.

For the treatment analysis, patients were divided into five large groups according to pathologies for better data correlation from a smaller number of variables: macular edema, MNV, neovascular glaucoma (NVG), preoperative indication (tractional retinal detachment caused by proliferative diabetic retinopathy), and tumors and vasculopathies.

The free R software was used in the statistical analyses^(19)^. Initially, descriptive analyses were performed to comprehend better the profile of patients, and the relationships between the variables were observed. Categorical variables are presented as total and relative frequencies, whereas continuous variables were presented as average, quartiles, and standard deviations. Regarding the statistical tests used, the chi-square adherence test (in the analysis of the variables referring to the general profile of patients and regarding pathologies, medications, and treatment regimens used), independent chi-square test (in the analysis of adherence and treatment outcomes per pathology), Fisher’s exact test (analysis of VA per pathology), and Kruskal-Wallis test were used (analysis of the number of IV injections per pathology or treatment regimen used). In this study, a 5% significance level was adopted, with p values <5% considered significant and highlighted with an asterisk, and values <0.1% were indicated with two (2) asterisks.

## RESULTS

A total of 446 patients received antiangiogenic injections at Hospital das Clínicas da Unicamp between January and December 2020. A total of 17 patients were excluded (death, n=7; missing medical records, n=10). A total of 429 patients and 514 eyes were analyzed.

All patients were treatment-naïve, and none developed endophthalmitis during follow-up or other serious complications related to the intravitreal application of anti-VEGF.

Most of the patients who were treated with antiangiogenic agents during the study period were male (51.28%) and white (80.89%), with a predominance of patients aged 60-70 years (33.8%) and those with complete or incomplete primary education (56.88%). A statistically significant difference was found (p<0.001), except in the sex analysis, where the hypothesis of equality between the categories cannot be rejected (p>0.05).

As regards the origin of the patients, 78.55% did not belong to the Regional Health Departament (RHD) VII to which the city of Campinas belongs (p<0.001), and the majority of the patients were admitted to the emergency department (66.67%).

Of the 514 eyes treated with antiangiogenic agents, most received Bevacizumab injection (79.38%), the principal treatment regimen used was pro re nata (90.27%), and a significant difference was found between the groups (p<0.001). The most common pathology group indicative of anti-VEGF therapy was macular edema (60.12%), with 48.25% of diabetic etiology and 11.09% secondary to venous occlusions, followed by MNV (21.4%) with exudative AMD accounting for 12.45% of the injected eyes.

The mean number of intravitreal injections needed per patient was 3.83. A significant difference (p<0.001) was found in the number of injections received by each pathology group and the treatment regimen used. The MNV group had the highest median (5 injections), and patients undergoing complete or incomplete PRN required fewer applications (median of 3 injections) than those following the treat and to extend (T&E, median of 6) or mixed (median of 7) regimens.

Regarding follow-up of the proposed treatment, nearly half of the patients (41.47%) did not adhere to the recommended scheme. Moreover, a dependent relationship was found between treatment adherence and pathology (p<0.001). More than 80% of the patients with NVG, preoperative indications, and tumors or vasculopathies adhere to the treatment regimen, whereas 60% of the patients with macular edema and neovascular macular membrane adhered to the treatment regimen.

In this study, the low mean value of intravitreal injections received per patient (3.83), equivalent only to the minimum loading dose used in most diseases, can be explained by the high rate of non-adherence to treatment. The average number of injections would be higher if patients followed the proposed treatment regimen.

As regards the outcomes of the antiangiogenic therapy, 20.04% of the patients stopped the injection by medical indication during follow-up, 45.29% continued the treatment, but 34.67% were lost to follow-up. However, no association was found between the treatment outcome and underlying pathology.

When assessing the functional response of the patients, most of them showed improvement (36.96%) or stability (31.32%) of their VA. No association was found between VA improvement and underlying pathology (p=0.078).

## DISCUSSION

Among the 429 patients treated with intravitreal antiangiogenic injections in 2020, 80% were ≥50 years old and 69% were >60 years old (mean age, 60.92 years; Table 1). The higher prevalence of this age group is justified by the finding that the main pathologies indicative of treatment with anti-VEGF injection in this study were DME (48.25%) followed by exudative AMD (12.25%), which are common in older groups (Table 2) .

**Table 1:** General profile of the patients who were treated with intravitreal antiangiogenics in Hospital das Clínicas at Unicamp

	Frequency			
Patients treated with antiangiogenics	N	%	95% CI	p-value
**Sex**				
Female	209	48.72	(43.91%, 53.55%)	**0.6**
Male	220	51.28	(46.45%, 56.09%)	
Total	429	100		
**Age group**				<0.001**
<50 years	46	10.72	(8.03%, 14.14%)	
50-60 years	87	20.28	(16.64%, 24.46%)	
60-70 years	145	33.8	(29.37%, 38.52%)	
70-80 years	100	25.64	(21.63% 30.1%)	
≥80 years	41	9.56	(7.02%, 12.84%)	
Total	429	100		
**Educational attainment**				<0.001**
No schooling	21	4.9	(3.13%, 7.5%)	
Complete or incomplete primary education	244	56.88	(52.03%, 61.6%)	
Complete or incomplete secondary education	81	18.88	(15.35%, 22.98%)	
Complete or incomplete college education	23	5.36	(3.5%, 8.05%)	
Not available	60	13.99	(10.92%, 17.72%)	
Total	429	100		
**Race**				<0.001**
White	347	80.89	(0.01%, 1.5%)	
Brown	59	13.75	(76.77%, 84.43%)	
Yellow	1	0.23	(10.71%, 17.46%)	
Black	22	5.13	(3.32%, 7.78%)	
Total	429	100		
**Belong to the RHD VII**				<0.001**
No	337	78.55	(75.04%, 82.93%)	
Yes	92	21.45	(17.07%, 24.96%)	
Total	429	100		

Footnotes: Chi-square adherence test was used.

CI= confidence interval; RHD= regional health department.

**Table 2:** Frequency of pathologies in eyes treated with intravitreal antiangiogenics

		Frequency			
Pathology group	Pathology subgroup	N	%	95% CI	p-value
**Macular edema**	Edema secondary to venous occlusion	57	11.09	(55.73%, 64.35%)	<0.001**
	Diabetic macular edema	248	48.25		
	Irvine-Gass syndrome	4	0.78		
	Total	309	60.12		
**Macular neovascularization**	Aneurysmal type 1 MNV	20	3.89	(17.98%, 25.25%)	0.43
	Neovascular AMD	64	12.45		
	Pachyochoroid neovascularization	11	2.14		
	MNV of undetermined origin	4	0.78		
	Type 3 MNV	2	0.39		
	Secondary to degenerative myopia	7	1.36		
	Secondary to angioid streaks	2	0.39		
	Total	110	21.4		
**Preoperatively**	Preoperatively	29	5.64	(3.88%, 8.1%)	<0.001**
**NVG**	NVG	60	11.67	(9.09%, 14.84%)	<0.001**
**Tumors and vasculopathies**	Intraocular metastasis	1	0.19	(0.48%, 2.65%)	<0.001**
	Coats disease	2	0.39		
	Choroidal hemangioma	2	0.39		
	Eales disease	1	0.19		
	Total	6	1.17		

Footnotes: Chi-square adherence test was used (p-value <0.001**).

AMD= age-related macular degeneration; CI= confidence interval; MNV= macular neovascularization; NVG= neovascular glaucoma.

These numbers were comparable to the findings of a study conducted in another public referral hospital in São Paulo, in which the most treated disease was diabetic maculopathy (55%), followed by AMD (23%)^(20)^. However, they differ from a survey conducted by the Brazilian Retina and Vitreous Society in 2015, in which AMD required numerous IV injections (57%), followed by DME (27%)^(21)^.

This difference can be justified by the difference between the socioeconomic characteristics of the populations analyzed because in the latter most of the patients came from private practices and perhaps had better lifestyle habits and glycemic control than patients who used the SUS, as assessed in the present study.

No significant difference was found between the sexes (Table 1) because most of the diseases commonly discovered in the study had no predominance in men or women.

As for race (Table 1), 80.89% declared themselves to be white. Given the two most common pathologies in the population investigated, such a result is expected in AMD, which is a risk factor for disease onset^(22)^. By contrast, national data show that the black race has the highest diabetes prevalence, which was not reflected in the study findings because it only accounted for 5.13% of the patients^(23)^.

As regards the place of birth (Table 1) 57.8% of the patients were born in the state of São Paulo. When divided by the RHD, the territory corresponding to RHD VII, Campinas and Epidemiological Surveillance Group XVII, is made up of 42 municipalities divided into four health regions: Metropolitana de Campinas, Circuito das Aguas, Jundia, and Bragança, with a population of 4,446,535, making it São Paulo’s third most populous RHD^(24)^.

Only 21.45% of the patients belonged to DRS VII, which is not consistent with the patient profile seen at Unicamp’s HC. Accordingly, when we examined the patients’ entrance doors, the majority of them (66.67%) arrived from the emergency department, which provides free care for patients with ophthalmologic symptoms. The huge number of patients who do not belong to the geographically proposed department can be explained by such statistics.

In terms of educational attainment (Table 1), 61.78% of the patients had completed primary school. This is consistent with the findings in the whole Brazilian population, which showed that among those aged ≥25 years, 46.6% have completed basic school or its equivalent, 27.4% have completed high school or its equivalent, and 17.4% have completed college^(25)^.

An agreement was also found regarding the higher frequency of diabetes mellitus in populations with low education levels in Brazil and other countries. Education is an important socioeconomic indicator and implies differentiated risks in health and disease, especially because of the vulnerable living environment, less access to health services, and poorer practices for eating, physical activity, body care, and disease prevention^(23)^.

The most commonly used medication was bevacizumab at HC Unicamp during the study period, corresponding to 79.38% of the injected eyes, which may be directly related to the greater availability of the medication in the service associated with its best cost-benefit (Table 3).

**Table 3:** Distribution of eyes treated with antiangiogenics according to medications and treatment regimens

	Frequency			
Patients treated with antiangiogenics	N%	%	95% CI	p-value
**Antiangiogenic medication**				<0.001**
Bevacizumab	408	79.38	(75.57%, 82.74%)	<0.001**
Ranibizumab	10	1.95	(0.99%, 3.67%)	<0.001**
Aflibercept	23	4.47	(2.92%, 6.74%)	<0.001**
Switch between antiangiogenics	42	8.17	(6.02%, 10.97%)	<0.001**
Antiangiogenics with com switch to corticosteroids	31	6.03	(4.2%, 8.55%)	<0.001**
Total	514	100		
**Used treatment regimen**				<0.001**
PRN complete or incomplete	464	90.27	(87.3%, 92.63%)	<0.001**
Mixed	19	3.7	(2.3%, 5.82%)	<0.001**
T&E	31	6.03	(4.2% 8.55%)	<0.001**
Total	514	100		
**Switch between medications**				<0.001**
No	441	85.8	(82.41%, 88.64%)	
Yes	73	14.2	(11.36%, 17.59%)	
Total	514	100		

Footnotes: Chi-square adherence test was used.

CI= confidence interval; PRN= pro re nata; T&E= treat and extend.

Bevacizumab is a monoclonal antibody against all isoforms of VEGF A, which has been approved by the FDA for intravenous use in the treatment of colorectal cancer. It is deemed effective in the treatment of the main pathologies in the study, providing both improvements in VA and a reduction in retinal thickness. For DME, DRCR.net has proved its efficacy, as have the CATT, IVAN, and GEFAL studies for exudative AMD. Despite its widespread use, it is considered an off-label treatment for retinal diseases^(20)^.

A PRN regimen was used to treat the vast majority of patients (90.27%) (Table 3). The PRN (or as needed) regimen includes a loading dosage of usually three intravitreal injections weekly, followed by monthly surveillance. Further injections are given if choroidal neovascularization, macular edema, hemorrhage, or impaired VA are present. The T&E regimen includes 4-week injections until maximum effect and a subsequent extension of the interval until the next treatment. This regimen is increasingly used for exudative AMD and other types of MNV such as polypoidal vasculopathy^(26)^. Patients who were treated as needed but without the usual three-dose loading were considered to have an incomplete PRN regimen, and patients who switched treatment strategies during follow-up were considered to follow a mixed regimen.

Considering the number of injections, each patient received an average of 3.83. For the analysis of the number of injections received by pathology or treatment regimen used, we considered the median number of injections because the mean is influenced by extreme values, which greatly hinders data analysis and interpretation.

**Figure 1:**
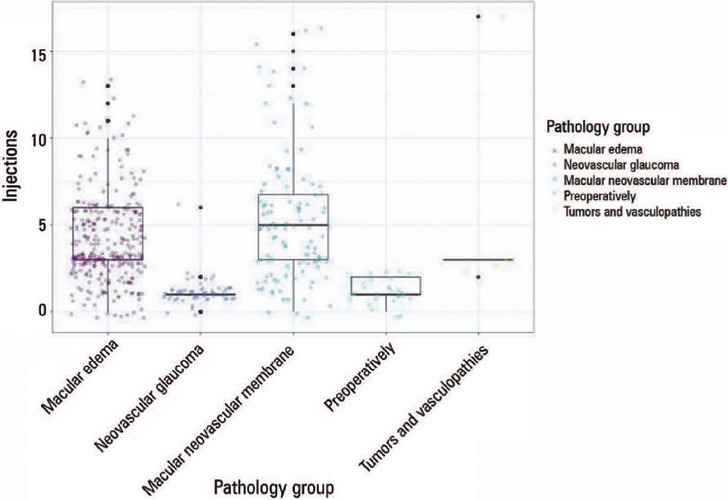
Distribution of the number of intravitreal injections by pathology group.

When correlating the number of injections to the pathologies indicative of treatment with anti-VEGF, MNV, and macular edema groups, which were led by chronic diseases AMD and DME, respectively, require a higher number of injections by the recurrence of activity^(27,28)^ (median injections of 5 and 3, respectively). This finding is different from those in patients with NVG and preoperatively where intravitreal injections are used as an emergency measure and usually show good response. In patients with tumors and vasculopathies, the analysis was restricted by the small sample size (Figure 1).

The Kruskal-Wallis test was used (p<0.001).

**Figure 2:**
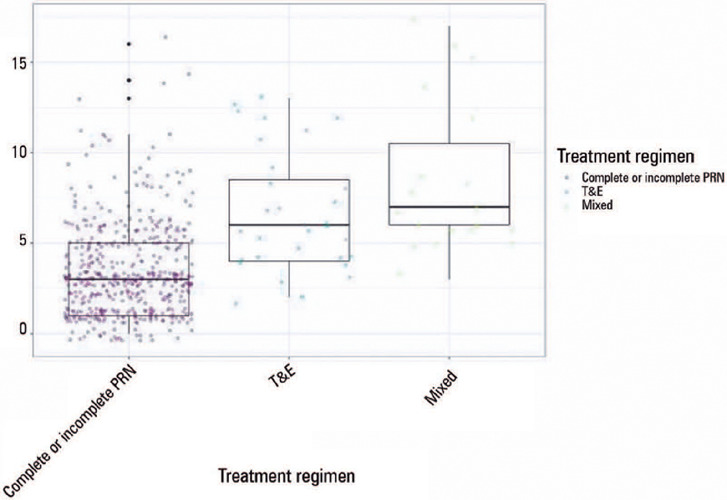
Distribution of the number of intravitreal injections by treatment regimen.

Regarding treatment regimens, the PRN regimen used a few number of injections than the T&E and mixed regimens, with mean values of 3 compared with 6 and 7, respectively (Figure 2). Based on the literature, the number of injections in the T&E Group was lower than that in the present study because this treatment regimen aims precisely at reducing the number of injections and visits. This group needs an increased number of injections because the proposed intervals do not always correspond to the latter, given the difficulty of scheduling and the limited number of antiangiogenic drugs in the service per month, which may have compromised the effectiveness of this treatment regimen.

The Kruskal-Wallis test was used (p<0.001).

PRN= pro re nata; T&E= treat and extend.

Considering the short follow-up time of the study and the reduced mean value of injections per patient (3.83), the 14.2% switching rate between medications was relatively high because normally more doses are needed before deciding to switch drugs. However, as this is a public service in which the availability of medications is limited, this high value may have been associated with the unavailability of medications and the need to replace the medication available at that time.

In the analysis of adherence to the proposed treatment (Table 4), a significant portion of the patients (41.47%) missed at least one scheduled intravitreal injection, not adhering to the recommended treatment. Non-compliance rates were considerably higher in the MNV and macular edema groups because they include more chronic diseases and, therefore, more vulnerable to missed appointments, as they almost always require more visits for evaluations and injections^(27,28)^.

**Table 4:** Frequency of adherence and treatment outcomes according to the pathology group

	Pathology group										
	Macular Edema		NVG		MNV		Preoperatively		Tumors and vasculopathies		
	N	%	N	%	N	%	N	%	N	%	p-value
**Adherence to treatment**											<0.001**
Yes	128	52.24	46	90.2	48	49.48	20	80	5	83.33	
No	117	47.76	5	9.8	49	50.52	5	20	1	16.67	
Total	245	100	51	100	97	100	25	100	6	100	
**Outcome of the therapy**											**0.117**
Lost to follow-up	90	36.73	16	31.37	35	36.08	5	20	1	16.67	
Continued in treatment	116	47.35	19	37.25	42	43.3	11	44	4	66.67	
Stopped the injection by medical indication	39	15.92	16	31.37	20	20.62	9	36	1	16.67	
Total	245	100	51	100	97	100	25	100	6	100	

Footnotes: Independent Chi-square test was used (for the analysis, five patients with different pathologies in both eyes were excluded).

NVG= neovascular glaucoma; MNV= macular neovascularization.

Another factor that may have corroborated why nearly half of the patients did not adhere to antiangiogenic treatment was the restrictions during the COVID-19 pandemic, which resulted in patients missing numerous ophthalmologic consultations and procedures. In agreement with such a hypothesis, an Italian study reported a reduction in intravitreal anti-VEGF injections by 48.5% and 48.6% during the pandemic when compared with the intra- and inter-annual control periods, respectively^(29)^.

In the functional response analysis (Table 5), approximately 70% showed VA improvement or stability, and only approximately 20% of the cases had worsened. No association was found between VA improvement and the underlying pathology. This analysis was possibly hampered by the small number of patients in certain groups, requiring higher studies. Although we cannot confirm a causal relationship, a limiting factor to VA improvement was the time interval between the complaint and the IV injection, which in this study was, on average, 10 months. Without proper treatment, this period may likely have contributed to the worsening of VA in some patients, considering that the delay in initiating antiangiogenic therapy is a factor of worse prognosis in most of the pathologies analyzed.

**Table 5:** Frequency of patients according to pathology in relation to visual acuity at baseline and last examination in 2020

Pathology group	Pathology subgroup	Stability	Improvement	Worsened	Not available
**Macular edema**	DME	78 (31.45%)	99 (39.92%)	42 (16.94%)	29 (11.69%)
	Irving-Gass syndrome	2 (50%)	1 (25%)	1 (25%)	0
	Secondary to venous occlusion	13 (22.81%)	25 (43.86%)	8 (14.04%)	11 (19.3%)
**NGV**	NGV	24 (40%)	12 (20%)	17 (28.33%)	7 (11.67%)
**MNV**	Wet AMD	22 (34.38%)	24 (37.5%)	14 (21.88%)	4 (6.25%)
	Aneurysmal type 1 MNV	6 (30%)	5 (25%)	6 (30%)	3 (15%)
	MNV of undetermined origin	1 (25%)	2 (50%)	0	1 (25%)
	Type 3 MNV	0	0	0	2 (100%)
	Degenerative myopia	2 (28.57%)	3 (42.86%)	1 (14.29%)	1 (14.29%)
	Pachychoroid neovascularization	2 (18.18%)	5 (45.45%)	2 (18.18%)	2 (18.18%)
	Angioid streaks	0	0	2 (100%)	0
**Preoperatively**	Preoperatively	10 (34.48%)	11 (37.93%)	5 (17.24%)	3 (10.34%)
	Coats disease	1 (50%)	1 (50%)	0	0
**Tumors or vasculopathies**	Eales disease	0	0	1 (100%)	0
	Choroidal hemangioma	0	1 (50%)	1 (50%)	0
	Intraocular metastasis	0	1 (100%)	0	0
Total	Total	161 (31.32%)	190 (36.96%)	100 (19.46%)	63 (12.26%)

Footnote: Fisher’s exact test was used (p-value =0.078).

DME= diabetic macular edema; MNV= macular neovascularization; NGV= neovascular glaucoma.

To reduce the waiting time for antiangiogenic therapy in the public health service, studies propose, among appropriate measures, better analysis of the indication for IV injections, such as in patients with no VA improvement even after several procedures. In addition, the creation of a waiting queue, according to the visual prognosis, would allow not only patients with a poor prognosis to continue treatment but also patients with diseases in early stages and with better prognosis to obtain faster access to anti-VEGF^(20)^.

This study evaluated the epidemiological and clinical profile of patients undergoing antiangiogenic therapy in a highly complex public hospital, which is fundamental for a better understanding of the demand for a reference ophthalmologic service in Brazil. Moreover, the analysis of functional results and user compliance profile makes it possible to optimize the indications and leverage the benefits of intravitreal therapy.

As a study limitation, the study had a short follow-up period. Therefore, studies with a longer follow-up are needed for a more conclusive analysis.

## References

[1] Avery RL, Bakri SJ, Blumenkranz MS, Brucker AJ, Cunningham ET Jr, DʼAmico DJ (2014). Intravitreal injection technique and monitoring: updated
guidelines of an expert panel. Retina.

[2] American Academy of Ophthalmology (2019). Age-related macular degeneration preferred practice
pattern[Internet].

[3] Gupta N, Mansoor S, Sharma A, Sapkal A, Sheth J, Falatoonzadeh P (2013). Diabetic retinopathy and VEGF. Open Ophthalmol J[Internet].

[4] Wells JA, Glassman AR, Ayala AR, Jampol LM, Aiello LP, Antoszyk AN, Arnold-Bush B, Baker CW, Bressler NM, Browning DJ, Elman MJ, Ferris FL, Friedman SM, Melia M, Pieramici DJ, Sun JK, Beck RW, Diabetic Retinopathy Clinical Research Network (2015). Aflibercept, bevacizumab, or ranibizumab for diabetic macular
edema. N Engl J Med.

[5] Tah V, Orlans HO, Hyer J, Casswell E, Din N, Sri Shanmuganathan V (2015). Anti-VEGF therapy and the retina: an update. J Ophthalmol [Internet].

[6] Qian T, Li X, Zhao M, Xu X (2018). Polypoidal choroidal vasculopathy treatment options: A
meta-analysis. Eur J Clin Invest.

[7] Andrés-Guerrero V, Perucho-González L, García-Feijoo J, Morales-Fernández L, Saenz-Francés F, Herrero-Vanrell R (2017). Current perspectives on the use of Anti-VEGF drugs as adjuvant
therapy in glaucoma. Adv Ther.

[8] Zhu Y, Zhang T, Xu G, Peng L (2016). Anti-vascular endothelial growth factor for choroidal
neovascularisation in people with pathological myopia. Cochrane Database Syst Rev[Internet].

[9] Sharma A, Bandello F, Loewenstein A, Kuppermann BD, Lanzetta P, Zur D (2020). Current role of intravitreal injections in Irvine Gass
syndrome-CRIIG study. Int Ophthalmol.

[10] Chatziralli I, Saitakis G, Dimitriou E, Chatzirallis A, Stoungioti S, Theodossiadis G (2019). ANGIOID STREAKS: a comprehensive review from pathophysiology to
treatment. Retina.

[11] Cheung CM, Lee WK, Koizumi H, Dansingani K, Lai TY, Freund KB (2019). Pachychoroid disease. Eye (Lond).

[12] Errera MH, Pratas A, Goldschmidt P, Sedira N, Sahel JA, Benesty J (2016). La maladie de Eales [Eales’ disease]. J Fr Ophtalmol.

[13] Sen M, Shields C, Honavar SG, Shields JA (2019). Coats disease: An overview of classification, management and
outcomes. Indian J Ophthalmol.

[14] Mathis T, Jardel P, Loria O, Delaunay B, Nguyen AM, Lanza F, Mosci C (2019). New concepts in the diagnosis and management of choroidal
metastases. Prog Retin Eye Res.

[15] Karimi S, Nourinia R., Mashayekhi A (2015). Circumscribed choroidal hemangioma. J Ophthalmic Vis Res.

[16] Solomon SD, Lindsley K, Vedula SS, Krzystolik MG, Hawkins BS (2019). Anti-vascular endothelial growth factor for neovascular age-
related macular degeneration. Cochrane Database Syst Rev.

[17] Papadopoulos N, Martin J, Ruan Q, Rafique A, Rosconi MP, Shi E (2012). Binding and neutralization of vascular endothelial growth factor
(VEGF) and related ligands by VEGF Trap, ranibizumab and
bevacizumab. Angiogenesis.

[18] Shiroma HF, Farah ME, Takahashi WY, Gomes AM, Goldbaum M, Rodrigues EB (2015). Survey: technique of performing intravitreal injection among
members of the Brazilian Retina and Vitreous Society (SBRV). Arq Bras Oftalmol.

[19] R Core Team (2021). R: A language and environment for statistical computing.

[20] Costa BM, Kawaguchi KY, Zaccaron BA, Shiguio LR (2020). Análise das injeções intravítreas do
Hospital do Servidor Público Estadual de São
Paulo. Rev Bras Oftalmol.

[21] Shiroma HF, Farah ME, Takahashi WY, Gomes AM, Goldbaum M, Rodrigues EB (2015). Survey: technique of performing intravitreal injection among
members of the Brazilian Retina and Vitreous Society (SBRV). Arq Bras Oftalmol.

[22] Connell PP, Keane PA, O’Neill EC, Altaie RW, Loane E, Neelam K, Nolan JM, Beatty S (2009). Risk factors for age-related maculopathy. J Ophthalmol.

[23] Malta DC, Szwarcwald CL (2019). Prevalência de diabetes mellitus determinada pela
hemoglobina glicada na população adulta brasileira, Pesquisa
Nacional de Saúde. Rev Bras Epidemiol [Internet].

[24] São Paulo, Governo do Estado, Secretaria da Saúde (2019). Plano de ação regional para o atendimento às
pessoas vítimas de acidentes por escorpião.

[25] IBGE educa Conheça o Brasil - população.

[26] Hufendiek K, Pielen A, Framme C (2018). Injektionsstrategien bei der Anwendung intravitrealer
VEGF-Inhibitoren: „Pro Re Nata versus Treat and Extend“ [Strategies of
Intravitreal Injections with Anti-VEGF: “Pro re Nata versus Treat and
Extend”]. Klin Monbl Augenheilkd.

[27] Bandello F, Battaglia Parodi M, Lanzetta P, Loewenstein A, Massin P, Menchini F, Veritti D (2017). Diabetic Macular Edema. Dev Ophthalmol.

[28] Mitchell P, Liew G, Gopinath B, Wong TY (2018). Age-related macular degeneration. Lancet.

[29] dell’Omo R, Filippelli M, Virgili G, Bandello F, Querques G, Lanzetta P, Avitabile T, Viola F, Reibaldi M, Semeraro F, Quaranta L, Rizzo S, Midena E, Campagna G, Costagliola C, Eyecare in Italy during COVID-19 pandemic (EICO) study
group (2022). Effect of COVID-19-related lockdown on ophthalmic practice in
Italy: A report from 39 institutional centers. Eur J Ophthalmol.

